# Mantle Cell Lymphoma Relapsing at the Lymphedematous Arm

**DOI:** 10.4084/MJHID.2013.016

**Published:** 2013-02-16

**Authors:** Giuseppina Massini, Stefan Hohaus, Francesco D’Alò, Valentina Bozzoli, Barbara Vannata, Luigi Maria Larocca, Luciana Teofili

**Affiliations:** 1 Departments of Hematology, Catholic University, Rome; 2 Departments of Pathology, Catholic University, Rome

## Abstract

Lymphedema (LE) is a chronic medical condition characterized by lymphatic fluid retention, resulting in tissue swelling. Cancer treatments involving lymph nodes can damage lymph drainage routes, causing accumulation of lymph fluid in the interstitial tissue of related limbs and body areas and secondary LE. Basically, the LE has a negative impact on physical and mental quality of life. Moreover, 0.07–0.04% of long term survivors (most patients undergoing mastectomy) can develop the Stewart-Treves syndrome, a rare and aggressive multifocal lymphangiosarcoma arising within the LE region. Here we describe the case of a 45-year-old woman with a massive LE of the left arm, as a consequence of previous breast cancer, who was diagnosed after 4 years of stage IV mantle cell lymphoma (MCL). The patient, after obtaining complete remission with chemotherapy and autologous hematopoietic stem cell transplant, had a relapse of MCL in the lymphedema site.

Lymphedema (LE) is a chronic medical condition characterized by lymphatic fluid retention, resulting in tissue swelling. Cancer treatments involving lymph nodes can damage lymph drainage routes, causing accumulation of lymph fluid in the interstitial tissue of related limbs and body areas and secondary LE. Recently, Paskett et al. reviewed risk factors, diagnosis and treatment of cancer related LE.^1^ The authors underlined that LE cannot be cured but it can only be managed in order to decrease limb size, improve limb function and prevent possible complications.^1^ Basically, the LE has a negative impact on physical and mental quality of life.^1^ Moreover, 0.07–0.04% of long term survivors (most patients undergone mastectomy) can develop the Stewart-Treves syndrome, a rare and aggressive multifocal lymphangiosarcoma arising within the LE region.^2^ The pathogenesis of this disease is not fully understood, even though a causative connection with chronic LE is evident.^2^ The differential diagnosis of Stewart Treves syndrome includes breast carcinoma metastatic to the skin, sarcoma of Kaposi, melanoma and cutaneous tumours other than lymphangiosarcomas.^2^ Overall, the prognosis Stewart-Treves syndrome is very poor, with a median survival of 2.5 years after diagnosis.^3^ The scarce delivery of antiproliferative drugs to the lymphedematous tissues most likely contributes to the resistance to chemotherapy observed these patients.^3^ The overall prevalence of LE has been estimated as 0.13–2% in industrialized countries.^4^ The number of patients with LE is expected to increase significantly over the next decades because of improved survival rates following cancer treatment. Indeed, it is conceivable that patients suffering from chronic LE would develop new cancer.

## Case report

**Figure 1:**
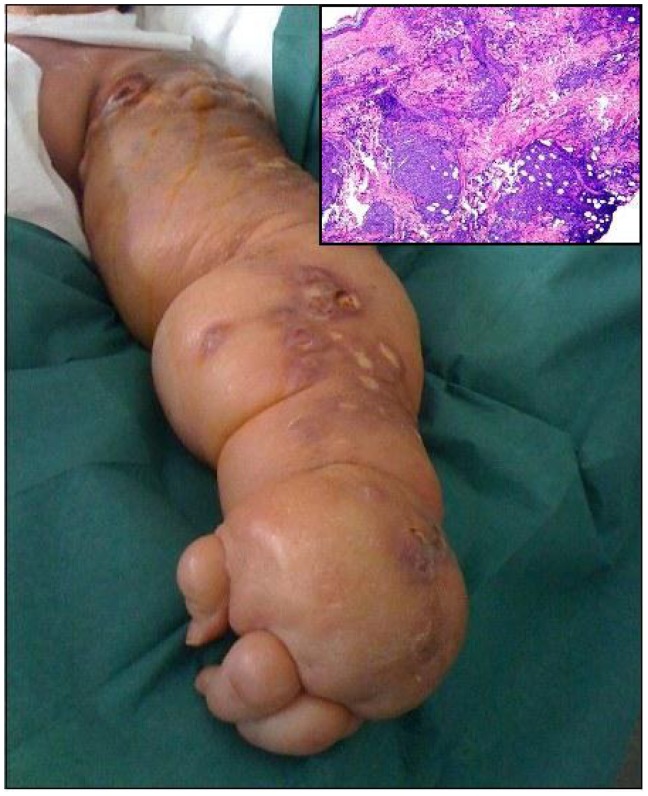
The photo shows the massive dissemination of cutaneous lesions over the entire arm surface, appearing as confluent nodules, partially ulcerated and infected. All lesions were confined to the region of LE. The biopsy revealed the multinodular infiltration of lymphoid cells (hematoxylin and eosin staining, 40× magnification).

A 45-year-old woman with stage IV mantle cell lymphoma (MCL) was recently admitted to our hematology department. She had a massive LE of the left arm, as a consequence of previous breast cancer, treated four years before with radical mastectomy, radiation and chemotherapy. The patient received four courses of R-CHOP (Rituximab 350 mg/m^2^ day 1, cyclophosphamide 750 mg/m^2^ iv day 1, doxorubicin 50 mg/m^2^ iv day 1, vincristine 1.4 mg/m^2^ ev days 1 and prednisone 1mg/m^2^ days 1–5), followed by three courses of R-MiCMA^5^ (Rituximab 350 mg/m^2^ day 1, Mitoxantrone 10 mg/m^2^ iv day 1, Carboplatin 100 mg/m^2^ iv days 1–4; Aracytin 2000 mg/m^2^ iv day 5 and Methylprednisolone 500 mg/m^2^ iv days 1–5) with haematopoietic stem cells (HSC) harvesting. The patient achieved complete remission and underwent autologous HSC transplantation as consolidation therapy. The post-transplant follow-up was uneventful for one year, when patient was again admitted to our department with a massive dissemination of purplish cutaneous nodules, in part ulcerated and infected, over the entire surface of the lymphedematous arm (Figure 1). She referred the appearance of small nodules on her left wrist two months before, which progressively sprouted over the entire arm without any systemic symptoms. The lesions were strictly confined to the area of chronic LE, reminding the clinical picture of the Stewart-Treves syndrome (Figure 1). A total body CT scan was performed, but no findings consisting with nodal or parenchymal lymphoma localizations were found. Moreover, bone marrow biopsy showed no lymphoma cell infiltration. The skin biopsy showed multinodular infiltration of lymphomatous cells (Figure 1) that at immunohistochemistry resulted CD20/CD5/CyclinD1positive, with a proliferative index of 90%. Patient received few courses of bortezomib and liposomal doxorubicin, without benefits; she died after few months with widespread pulmonary infiltration by MCL.

## Discussion

There are few cases reported in the literature on malignant lymphomas arising in a lymphoedematous arm, which can sometimes simulate the Stewart-Treves syndrome.^6^ The awareness of this possibility might help in the differential diagnosis of a suspicious cutaneous lesion within the LE. The MCL, in particular, is characterized by the striking tendency to disseminate throughout the body, infiltrating the lymphoid tissues, bone marrow and extranodal sites.^7^ Indeed, the aggressive clinical course observed in our patient is not surprising. However, we would like to stress that, at relapse, our patient had no localizations further than those in the lymphedematous arm. This finding suggests that LE could have favoured the persistence of an occult localization of lymphoma, despite routine investigations evidenced an apparent complete remission of disease. Actually, the impaired lymphatic drainage could have rendered the involved area like a “pharmacologic sanctuary”, allowing lymphoma cells to skip the antiproliferative effect of chemotherapy. In addition, the eventual local immunodeficiency, according to the hypothesis of the “immunocompromised district”, might have promoted the chemotherapy resistance of malignant cells.^8^,^9^

In conclusion, our experience emphasizes that, in assessing the response to chemotherapy of lymphoma patient with chronic LE, in addition to routine examinations, it should be performed a careful investigation of the involved extremity tailored to exclude residual tumour localizations.
